# Magnetic resonance imaging of the normal dromedary camel tarsus

**DOI:** 10.1186/s12917-021-02811-2

**Published:** 2021-03-02

**Authors:** Zakriya Ali Al Mohamad, Usama Hagag, Mohamed Gomaa Tawfiek, Ayman El Nahas

**Affiliations:** 1 Department of Clinical Sciences, College of Veterinary Medicine, King Faisal University, PO Box 400, 31982 Al-Ahasa, Kingdom of Saudi Arabia; 2 Department of Surgery, Anesthesiology and Radiology, Faculty of Veterinary Medicine, Beni-Suef University, 62511 Beni-Suef, Egypt; 3 Department of Anatomy and Embryology, Faculty of Veterinary Medicine, Beni-Suef University, 62511 Beni, Suef, Egypt

**Keywords:** Magnetic resonance imaging, Tarsus, Camel

## Abstract

**Background:**

Magnetic resonance imaging (MRI) is the most versatile and informative imaging modality for the diagnosis of locomotor injuries in many animal species; however, veterinary literature describing the MRI of the dromedary camel tarsus is lacking. Our purpose was to describe and compare the MRI images of twelve cadaveric tarsi, examined in a 1.5 Tesla MRI scanner, with their corresponding anatomical gross sections. Turbo spin-echo (TSE) T1-weighted (T1), T2-weighted (T2), proton density-weighted (PD), and short tau inversion recovery (STIR) sequences were obtained in 3 planes. Tarsi were sectioned in sagittal, dorsal, and transverse planes. MRI images from different sequences and planes were described and compared with the anatomical sections.

**Results:**

The soft and osseous tissues of the dromedary camel tarsus could be clearly defined on MRI images and corresponded extensively with the gross anatomic sections. The obtained MRI images enabled comprehensive assessment of the anatomic relationships among the osseous and soft tissues of the camel tarsus. Several structure were evaluated that cannot be imaged using radiography or ultrasonography, including the transverse inter-tarsal ligaments, the talocalcaneal ligament, the short dorsal ligament, branches of the short medial and lateral collateral ligaments and the tarsometatarsal ligaments. Specific anatomical features regarding the dromedary camel tarsus were identified, including the fused second and third tarsal bone, an additional bundle of the short medial collateral ligament connecting the talus and metatarsus and the medial and lateral limbs of the long plantar ligament.

**Conclusions:**

MRI images provided a thorough evaluation of the normal dromedary camel tarsus. Information provided in the current study is expected to serve as a basis for interpretation in clinical situations.

## Background

The camel tarsus is an anatomically complex region comprising numerous osseous and soft tissue structures [1]. The biomechanics of the tarsal region and the influence of loading on those structures are also complex [2]. Imaging of the tarsus with the commonly used diagnostic methods (radiography and ultrasonography) is technically a challenge due to the increased possibility of structural superimposition [3]. Indecisive outcomes, in complicated cases, with radiography and/or ultrasonography necessitate the use of advanced imaging techniques. Fortunately, Magnetic resonance imaging (MRI) allows for volumetric imaging of thin section views into multiple customizable planes, which enable the assessment of joint structures without superimposition [4]. MRI is the most sensitive noninvasive imaging method currently available for imaging of soft tissue structures [5], due to the superior contrast among different soft tissues without the use of ionizing radiation [6]. In horses, MRI can demonstrate lesions that are underestimated or undetected by radiography and/or ultrasonography [7] and enables the assessment of various tissues in greater anatomical and physiological details than do radiography and ultrasonography [8].

Hind limb lameness due to tarsal lesions is common and affects many breeds and disciplines [9]. MRI of the tarsus in horses has been shown to be specific and sensitive for detection of all types of conditions concerning soft tissues [9, 10] and osseous lesions [5] as well as detection of articular surface alterations through quantitative assessment of the osteochondral tissue and subchondral bone thickness [11]. However, MRI interpretation and lesion identification require experience with cross-sectional imaging and signal variations in normal animals in order to properly evaluate clinical cases and reach a definitive diagnosis and prognosis [9]. The normal MRI of the tarsus in the horse, cattle, cat, and the dog has been reported [3, 12–16] and computed tomography of the dromedary camel tarsus has been described [17]; however, no available data elucidating the normal MRI of the dromedary camel tarsus exist. Accordingly, the objective of the present study was to describe the normal MRI appearance of the dromedary camel tarsus with the aid of anatomical cryosections.

## Results

**Fig. 1:**
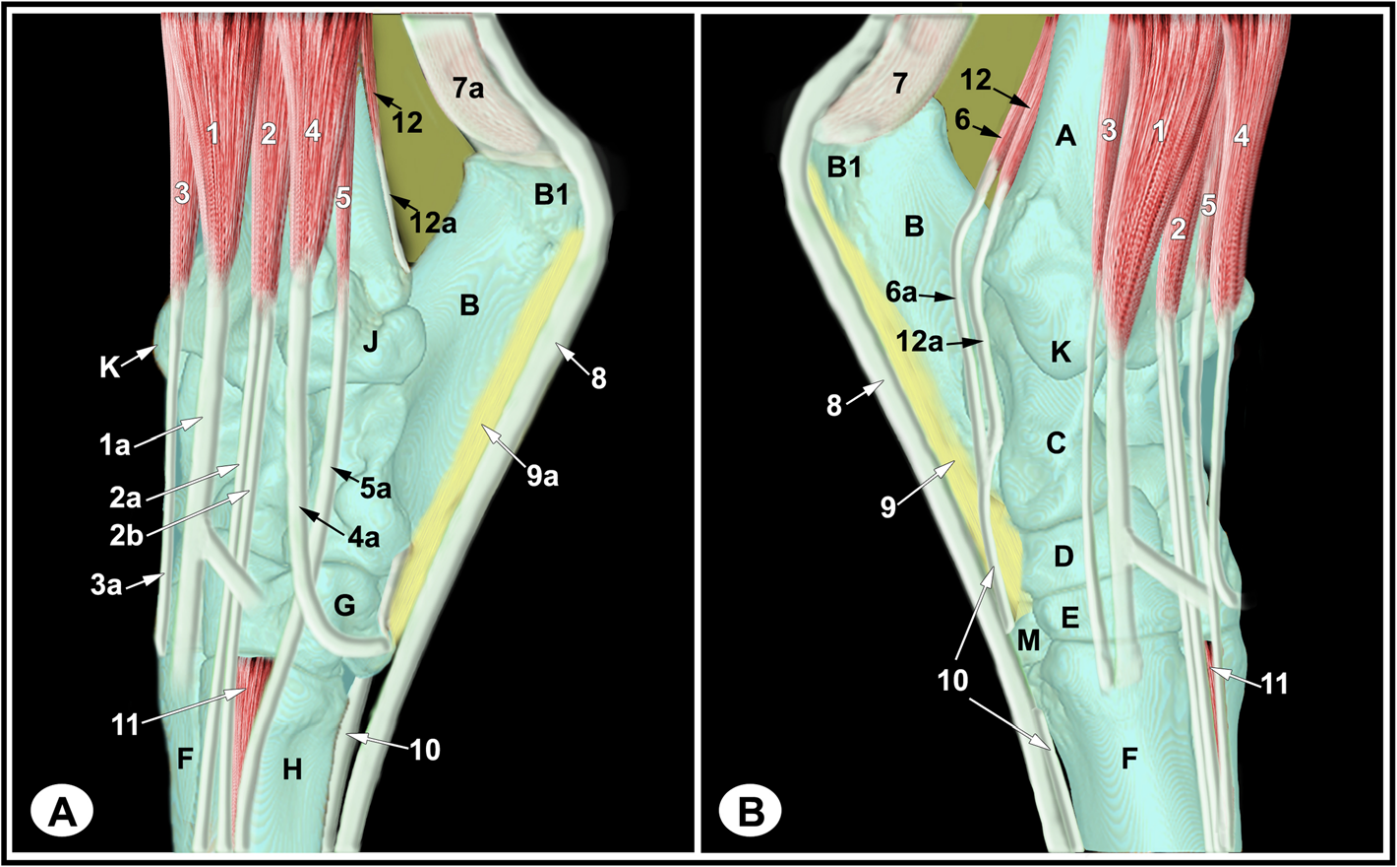
Muscles and tendons of the left dromedary camel tarsus (schematic, dorsolateral **a**, dorsomedial **b**). A, distal tibia; B, calcaneus; B1, calcaneal tuber; C, talus; D, central tarsal bone; E, fused 2nd and 3rd tarsal bone; F, 3rd metatarsal bone; G, 4th tarsal bone; H, 4th metatarsal bone; J, malleolar bone; K, medial malleolus; M, 1st tarsal bone; 1, fibularis tertius muscle; 1a, fibularis tertius tendon; 2, long digital extensor muscle ; 2a, medial digital extensor tendon (extensor of digit III); 2b, common digital extensor tendon (extensor of digit III and IV); 3, cranial tibial muscle; 3a, cranial tibial tendon; 4, fibularis longus muscle; 4a, fibularis longus tendon; 5, lateral digital extensor (LDE) muscle; 5a, LDE tendon; 6, medial digital flexor muscle; 6a, medial digital flexor tendon ; 7, medial tendon of gastrocnemius muscle; 7a, lateral tendon of gastrocnemius muscle; 8, superficial digital flexor tendon (SDFT); 9, long plantar ligament (LPL), medial limb ; 9a, lateral limb of LPL; 10, deep digital flexor tendon (DDFT); 11, short digital extensor muscle; 12, caudal tibial and lateral digital flexor muscles; 12a, common tendon of caudal tibial and lateral digital flexor muscles

**Fig. 2:**
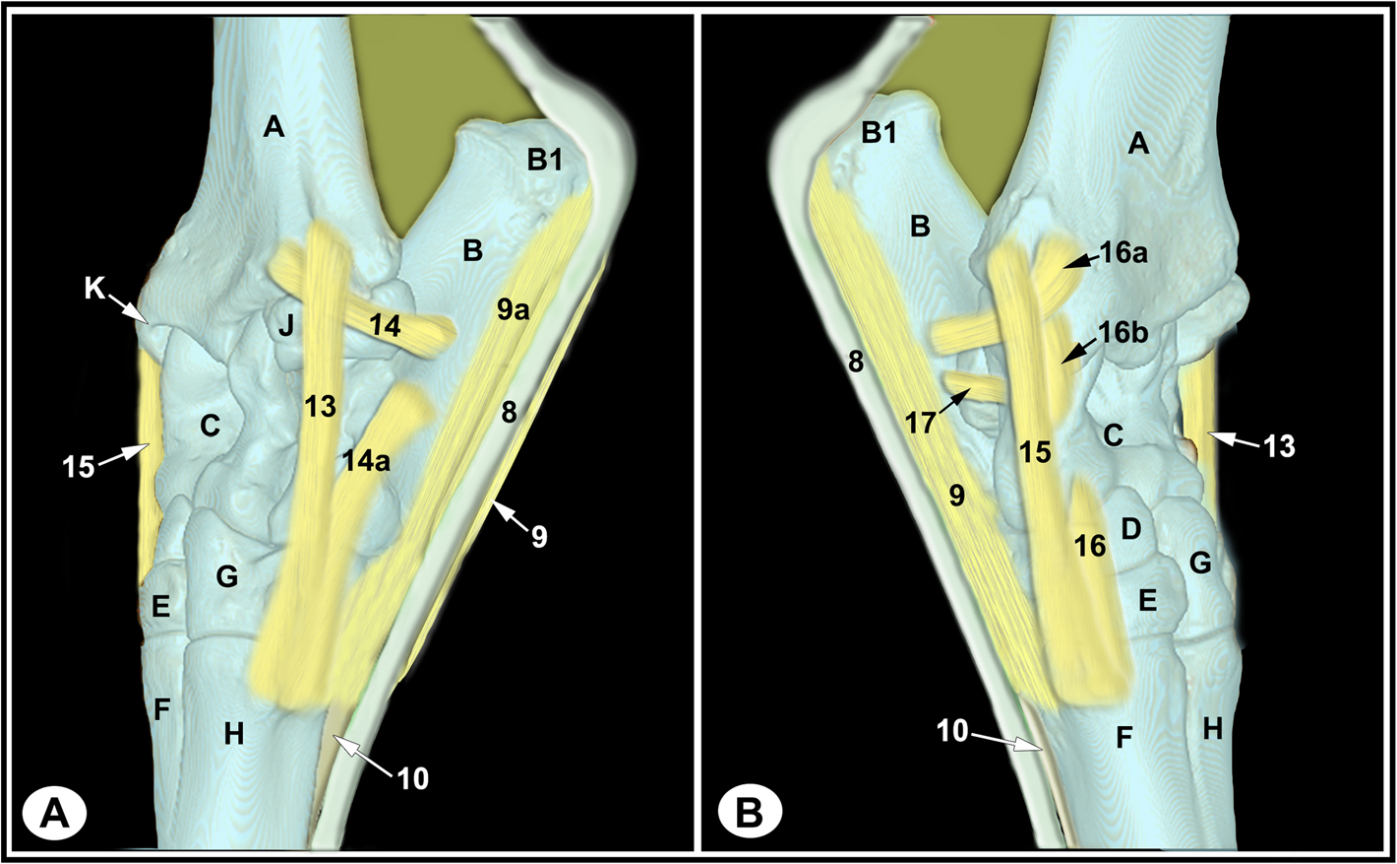
Ligaments of the left dromedary camel tarsus (schematic, dorsolateral **a**, dorsomedial **b**). A, distal tibia; B, calcaneus; B1, calcaneal tuber; C, talus; D, central tarsal bone; E, fused 2nd and 3rd tarsal bone; F, 3rd metatarsal bone; G, 4th tarsal bone; H, 4th metatarsal bone; J, malleolar bone; K, medial malleolus; 8, SDFT; 9, medial limb of LPL; 9a, lateral limb of LPL; 10, DDFT; 13, lateral collateral tarsal ligament (LCL), long part; 14, tibiocalcaneal branch of the short LCL; 14a, calcaneometatarsal branch of the short LCL; 15, medial collateral tarsal ligament (MCL), long part; 16, short MCL; 16a, tibiocalcaneal branch of short MCL; 16b, tibiotalar branch of short MCL; 17, talocalcaneal ligament

**Fig. 3:**
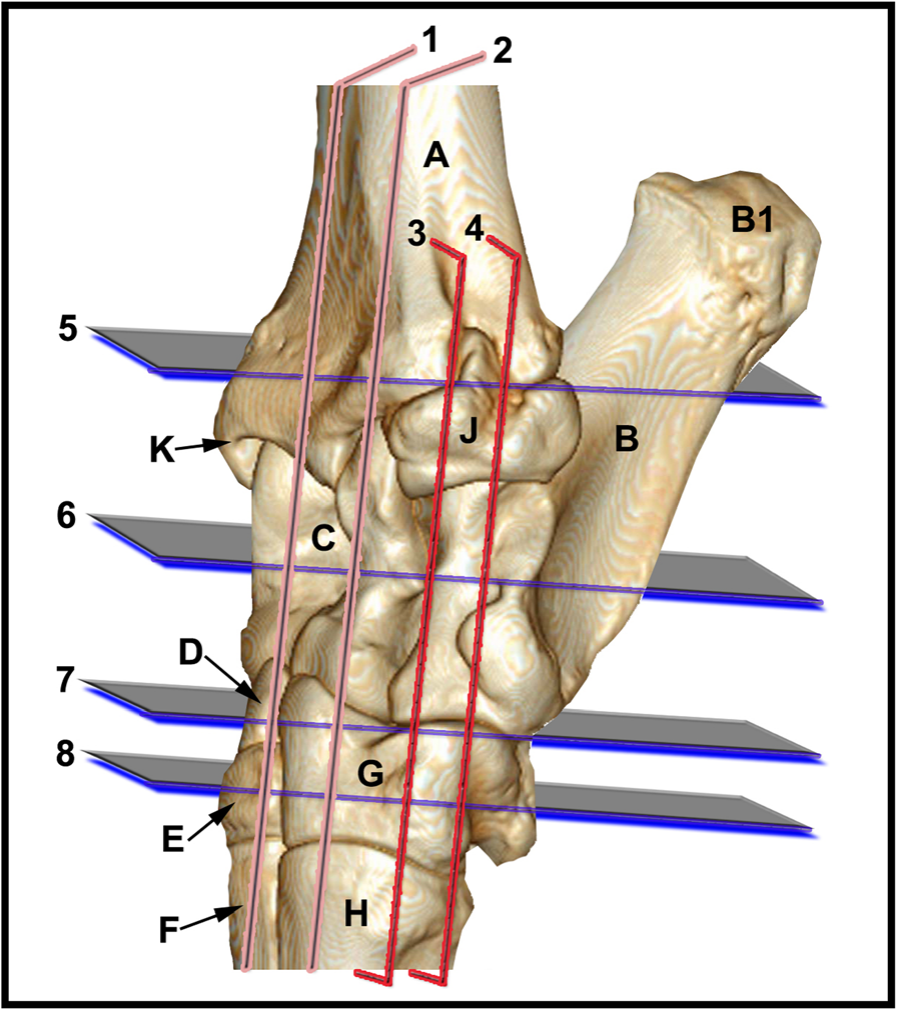
Medial sagittal T1-weighted MRI image (**a**) and gross anatomic section (**b**): at the level of the medial trochlear ridge of the talus, level 1 indicated in Fig. 11. A, tibia, cortical bone; A1, tibia, medullary cavity; A2, tibia, cancellous bone; B, calcaneus; B1, calcaneal tuber; B3, sustentaculum tali; C, talus; D, central tarsal bone; E, fused 2nd and 3rd tarsal bone; F, 3rd metatarsal bone; 1a, fibularis tertius tendon; 2, long digital extensor muscle; 3, cranial tibial muscle; 7, medial tendon of gastrocnemius muscle; 8, SDFT; 9, medial limb of LPL; 10, DDFT; 11, short digital extensor muscle; 12a, common tendon of caudal tibial and lateral digital flexor muscles; 18, tarsocrural joint; 19, plantar recess of the tarsocrural joint; 20, dorsal recess of the tarsocrural joint; 21, dorsal strengthening of tarsocrural joint capsule; 22, deep crural fascia; 23, proximal inter-tarsal joint; 24, inter-tarsal ligament; 25, tarsometatarsal joint

**Fig. 4:**
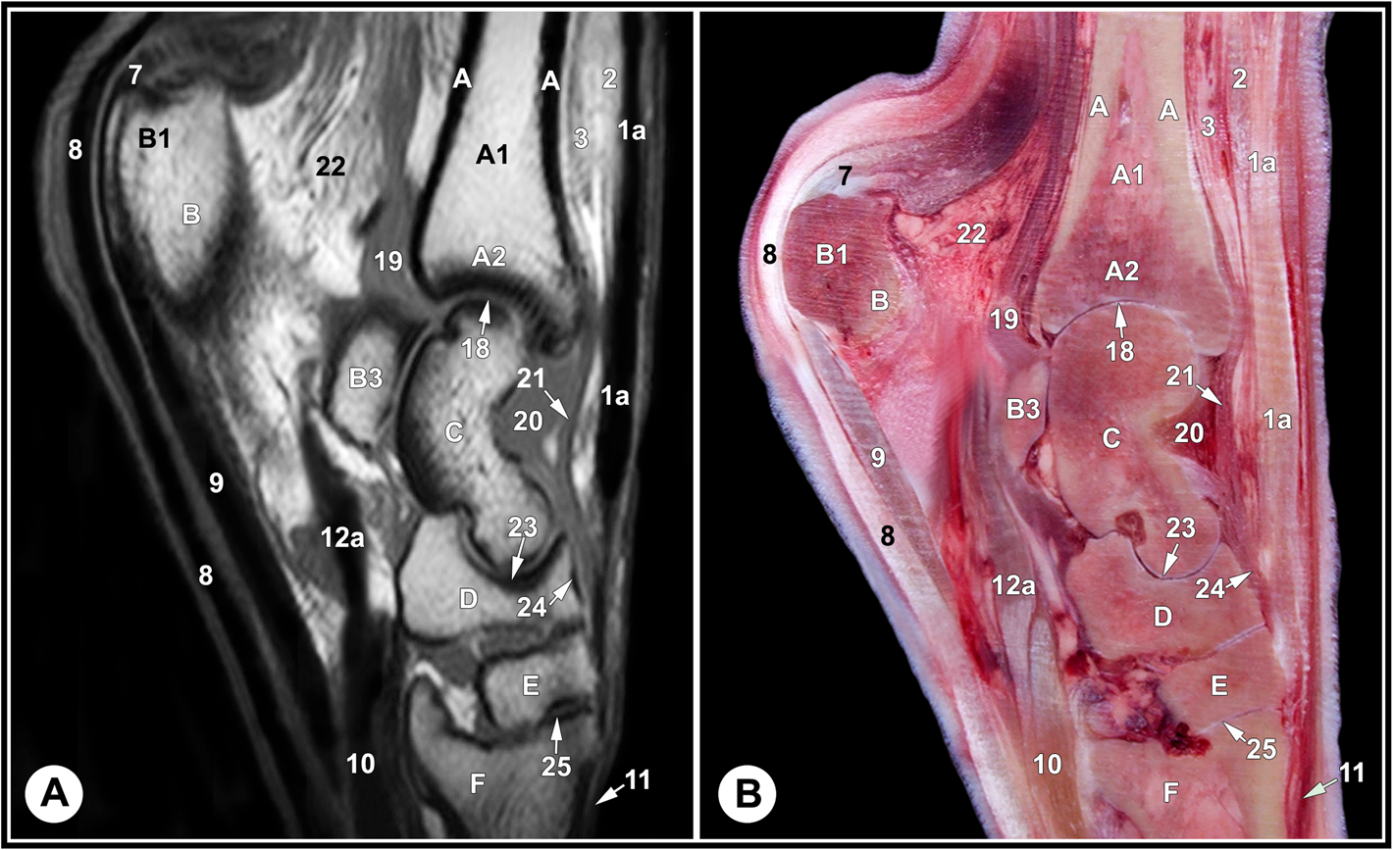
Lateral sagittal T2-weighted (**a**) and STIR (**b**) MRI images and gross anatomic section (**c**): at the level of the lateral trochlear ridge of the talus, level 2 indicated in Fig. 11. A, tibia, cortical bone; A1, tibia, medullary cavity; A2, tibia, cancellous bone; A3, tibia, cranial end of cochlea; B, calcaneus; B1, calcaneal tuber; B2, coracoid process; B4, calcaneus, bone marrow; C, talus; C1, proximal trochlea of the talus; C2, distal trochlea of the talus; G, 4th tarsal bone; H, 4th metatarsal bone; 1a, fibularis tertius tendon; 2, long digital extensor muscle; 2a, medial digital extensor tendon; 2b, common digital extensor tendon; 8, SDFT; 9a, lateral limb of LPL; 10, DDFT; 11, short digital extensor muscle; 18, tarsocrural joint; 19, plantar recess of tarsocrural joint; 20, dorsal recess of tarsocrural joint; 21, dorsal strengthening of tarsocrural joint capsule; 22, deep crural fascia; 23, proximal inter-tarsal joint; 25, tarsometatarsal joint; 26, crural extensor retinaculum

**Fig. 5:**
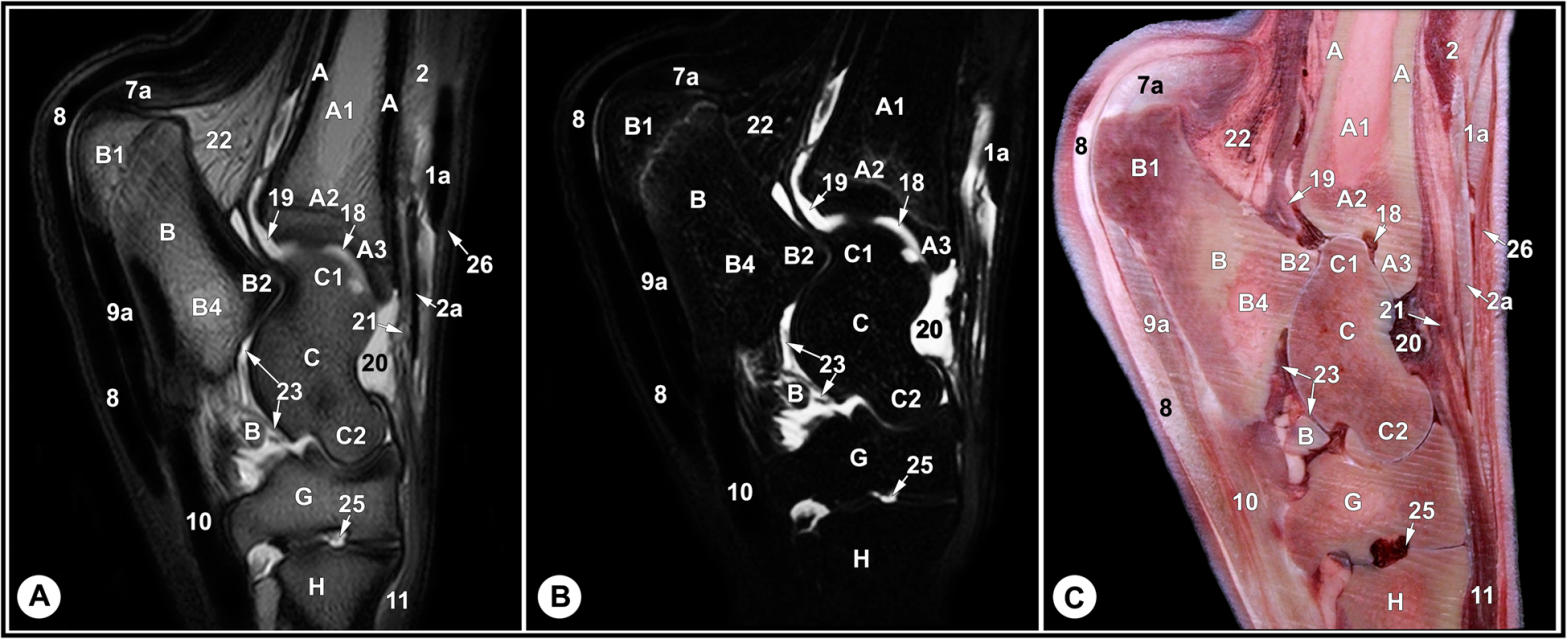
Cranial dorsal T1-weighted MRI (**a**) and gross anatomic section (**b**), level 3 indicated in Fig. 11. A, tibia, cortical bone; A1, tibia, medullary cavity; A2, tibia, cancellous bone; A4, tibial sagittal ridge; B, calcaneus; C, talus; C3, lateral part of the proximal trochlea of the talus; C4, medial part of the proximal trochlea of the talus; C5, lateral part of the distal trochlea of the talus; C6, medial part of the distal trochlea of the talus; C7, tarsal sinus; D, central tarsal bone; E, fused 2nd and 3rd tarsal bone; F, 3rd metatarsal bone; G, 4th tarsal bone; H, 4th metatarsal bone; J, malleolar bone; K, medial malleolus; 13, long LCL;14, tibiocalcaneal branch of the short LCL; 14a, calcaneometatarsal branch of the short LCL; 15, long MCL; 16, short MCL; 16a, tibiocalcaneal branch of the short MCL; 16b, tibiotalar branch of the short MCL; 17, talocalcaneal ligament.18, tarsocrural joint; 20, dorsal recess of the tarsocrural joint; 24, inter-tarsal ligament; 25, tarsometatarsal joint; 27, tarsometatarsal ligament

**Fig. 6:**
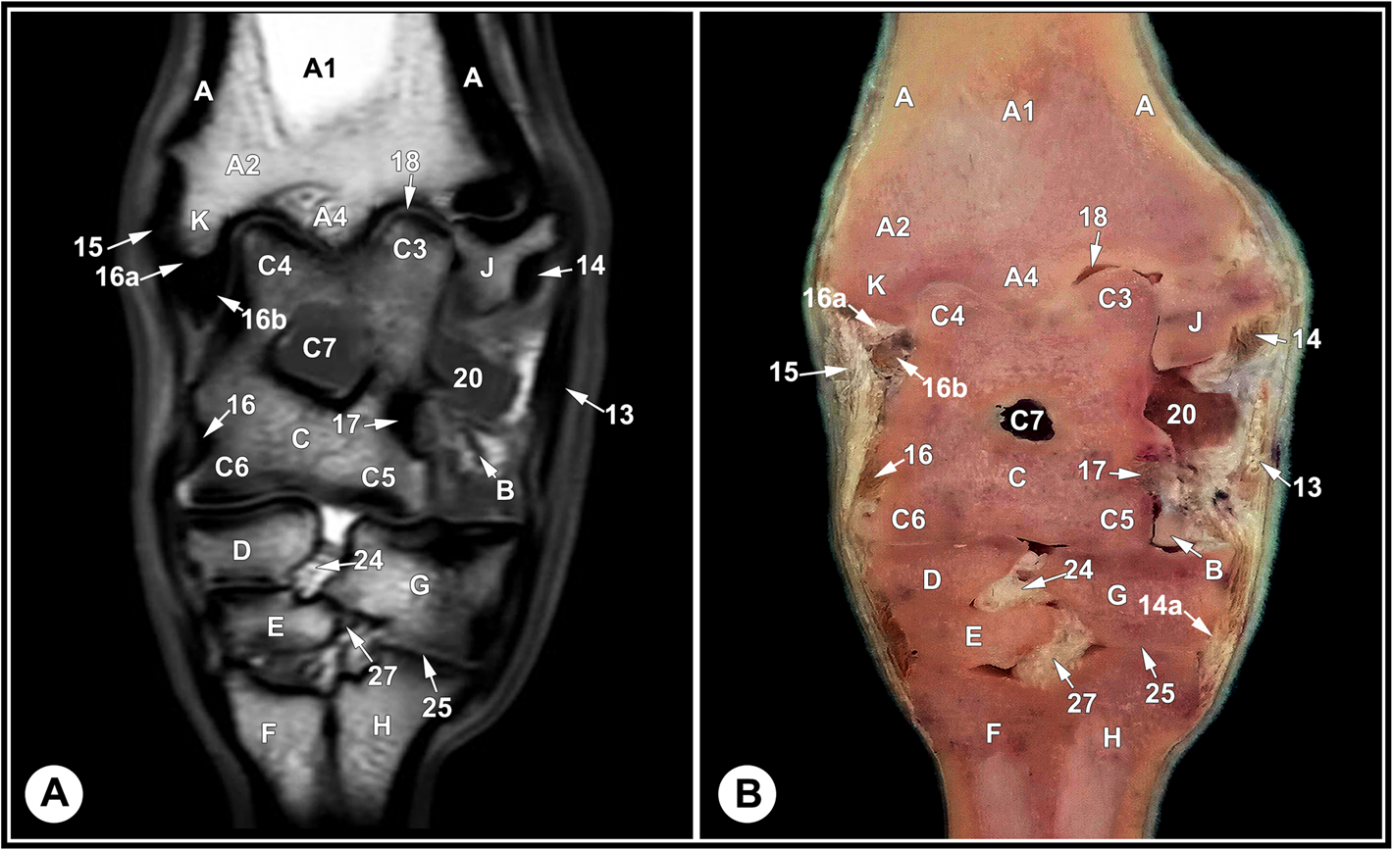
Caudal dorsal T2-weighted MRI image (**a**) and gross anatomic section (**b**), level 4 indicated in Fig. 11. A, tibia, cortical bone; A2, tibia, cancellous bone; B, calcaneus; C, talus; C3, lateral part of the proximal trochlea of the talus; C4, medial part of the proximal trochlea of the talus; C5, lateral part of the distal trochlea of the talus; C6, medial part of the distal trochlea of the talus; D, central tarsal bone; F, 3rd metatarsal bone; G, 4th tarsal bone; H, 4th metatarsal bone; J, malleolar bone; K, medial malleolus; M, 1st tarsal bone; 13, long LCL; 14, tibiocalcaneal branch of the short LCL; 14a, calcaneometatarsal branch of the short LCL; 15, long MCL; 16, short MCL; 16b, tibiotalar branch of the short MCL; 17, talocalcaneal ligament; 18, tarsocrural joint; 24, inter tarsal ligament

**Fig. 7:**
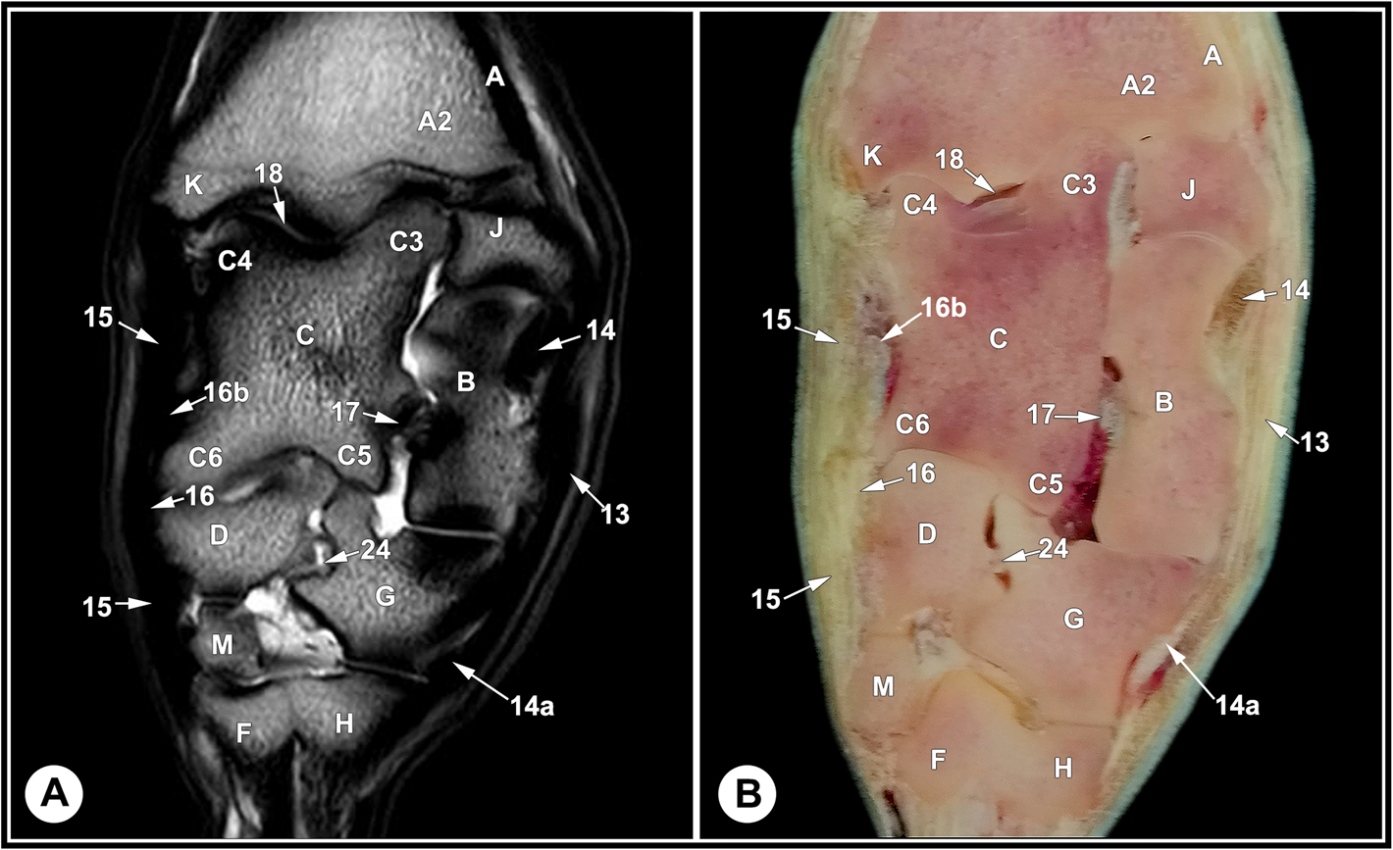
Transverse T1-weighted MRI image (**a**) and gross anatomic section (**b**): at the level of the distal tibia, level 5 indicated in in Fig. 11. A, tibia, cortical bone; A1, tibia, medullary cavity; A2, tibia, cancellous bone; B, calcaneus; B1, calcaneal tuber; J, malleolar bone; 1a, fibularis tertius tendon; 2a, medial digital extensor tendon; 2b, common digital extensor tendon ; 3a, cranial tibial tendon; 4a, fibularis longus tendon; 5a, LDE tendon; 6a, medial digital flexor tendon; 8, SDFT; 12a, common tendon of caudal tibial and lateral digital flexor muscles; 13, long LCL; 14, tibiocalcaneal branch of the short LCL; 15, long MCL; 22, deep crural fascia; 26, crural extensor retinaculum; 28, caudal branches of the saphenous artery and medial saphenous vein; 29, deep fibular nerve; 30, cranial tibial artery

**Fig. 8:**
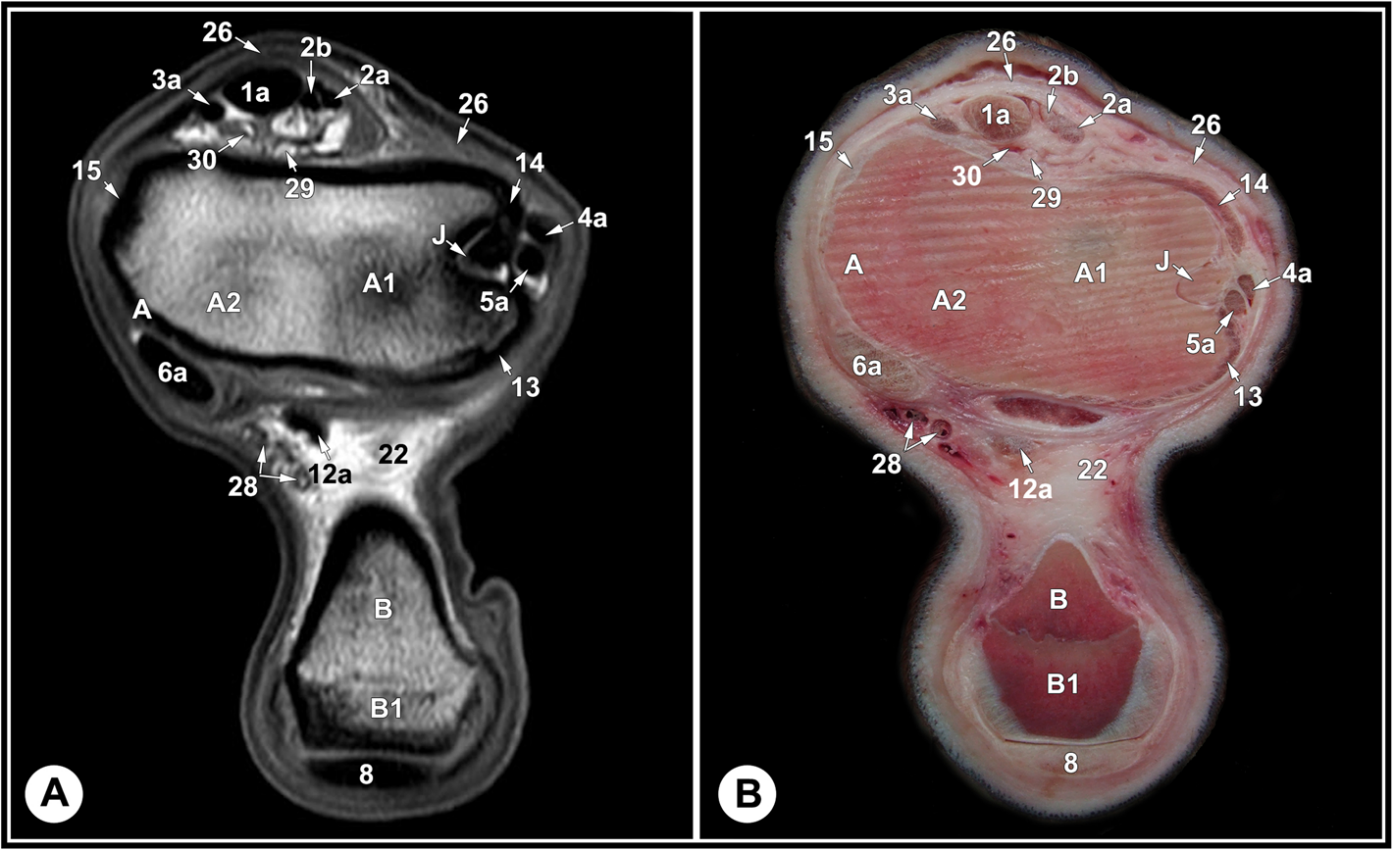
Transverse T1-weighted (**a**) and T2-weighted (**b**) MRI images and gross anatomic section (**c**): at the level of the trochlear ridges of the talus, level 6 indicated in Fig. 11. B, calcaneus; B4, calcaneus, bone marrow; B5, calcaneus, cancellous bone; C, talus; C3, lateral part of the proximal trochlea of the talus; C4, medial part of the proximal trochlea of the talus; 1a, fibularis tertius tendon; 2a, medial digital extensor tendon; 2b, common digital extensor tendon; 3a, cranial tibial tendon; 4a, fibularis longus tendon; 5a, LDE tendon; 6a, medial digital flexor tendon; 8, SDFT; 9, medial limb of LPL; 9a, lateral limb of LPL; 12a, common tendon of caudal tibial and lateral digital flexor muscles; 13, long LCL; 14, tibiocalcaneal branch of the short LCL; 14a, calcaneometatarsal branch of the short LCL; 15, long MCL; 16b, tibiotalar branch of the short MCL; 20, dorsal recess of the tarsocrural joint; 21, dorsal strengthening of tarsocrural joint capsule; 26, crural extensor retinaculum; 30, caudal branches of the saphenous artery and medial saphenous vein

**Fig. 9:**
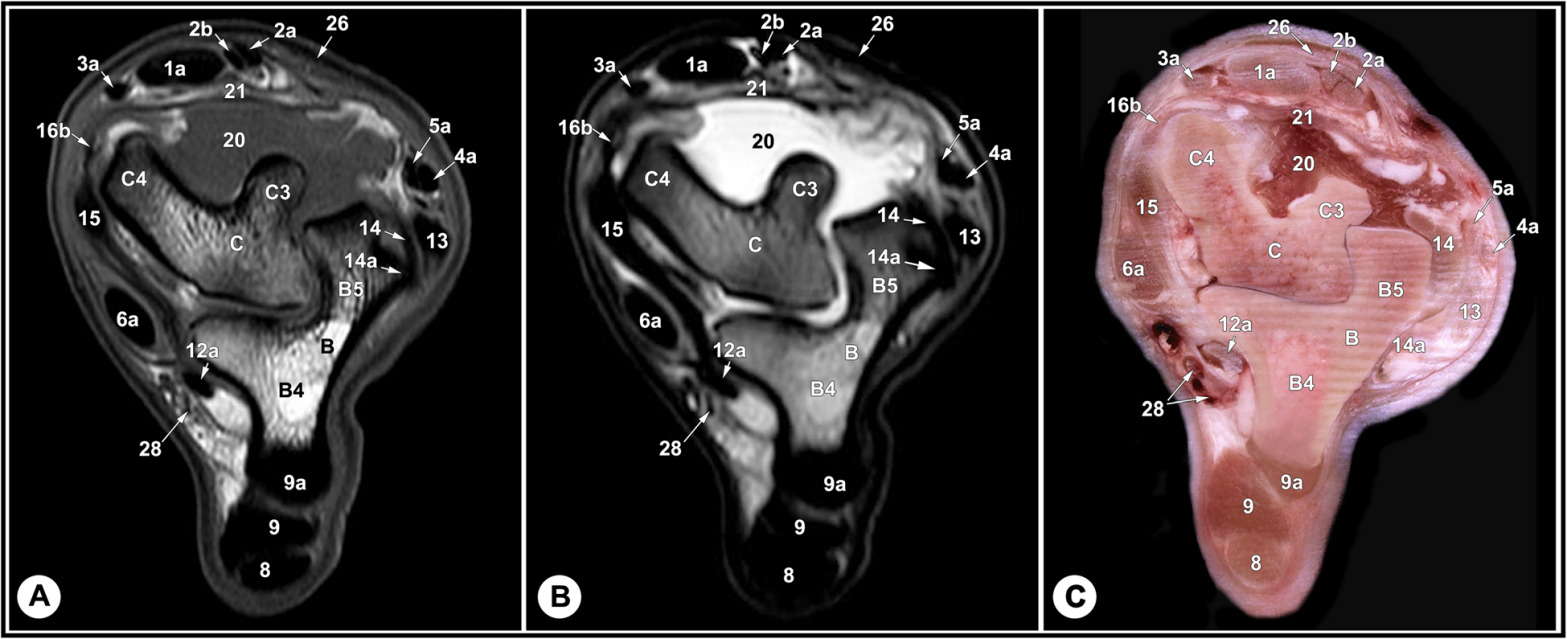
Transverse T1-weighted MRI image (**a**) and gross anatomic section (**b**): at the level of the proximal row of tarsal bones, level 7 as indicated in Fig. 11. D, central tarsal bone; G, 4th tarsal bone; 1a, fibularis tertius tendon; 1b, medial tendon of fibularis tertius muscle; 2a, medial digital extensor tendon; 2b, common digital extensor tendon; 3a, cranial tibial tendon; 4a, fibularis longus tendon; 5a, LDE tendon; 6a medial digital flexor tendon; 8, SDFT; 9, medial limb of LPL; 9a, lateral limb of LPL; 11, short digital extensor muscle; 12a, common tendon of caudal tibial and lateral digital flexor muscles; 13, long LCL; 14a, calcaneometatarsal of the short LCL; 15, long MCL; 16, short MCL; 31, plantar tarsal sheath

**Fig. 10:**
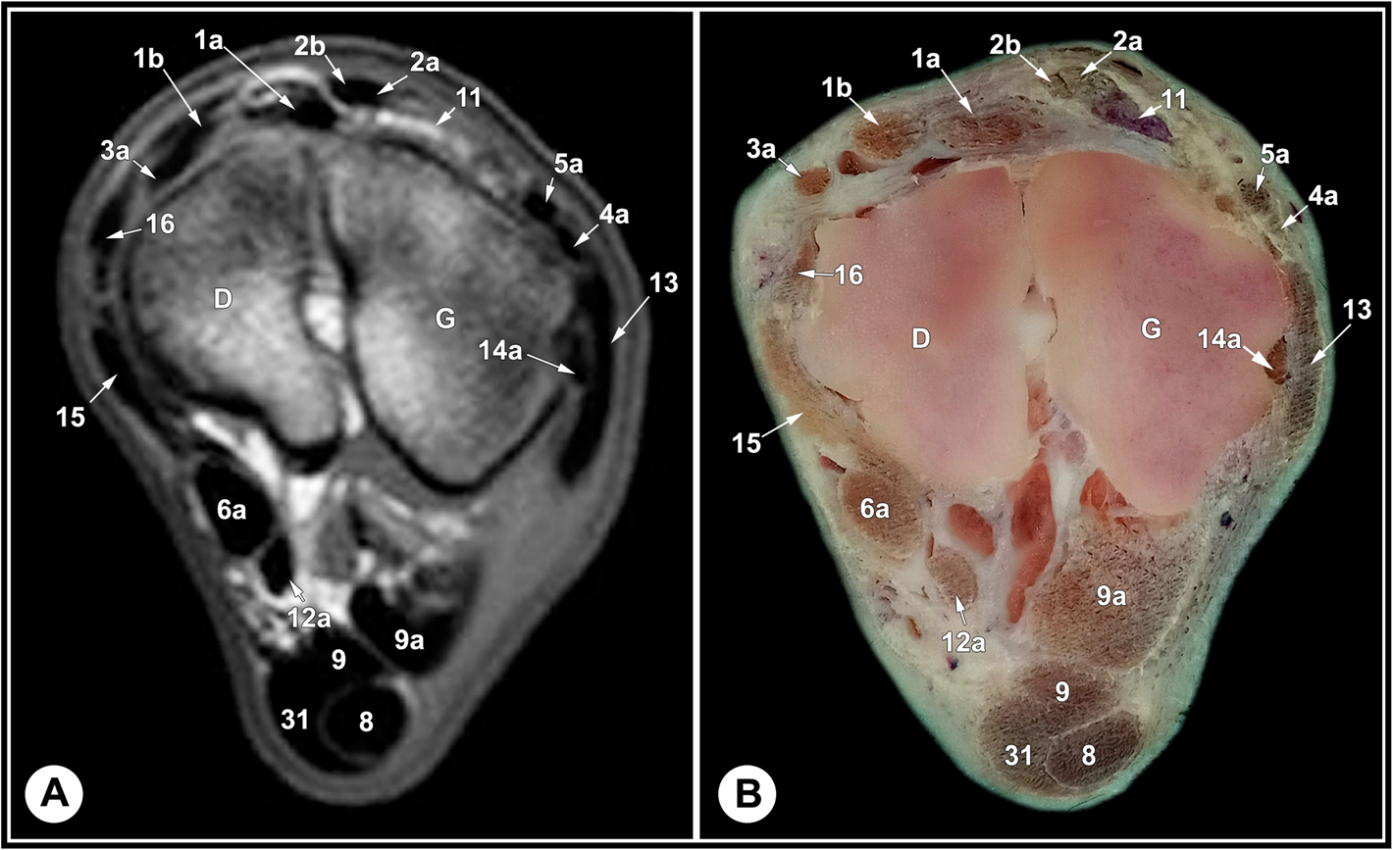
Transverse T2-weighted MRI image (**a**) and gross anatomic section (**b**): at the level of the distal row of tarsal bones, level 8 indicated in in Fig. 11. E, fused 2nd and 3rd tarsal bone; G, 4th tarsal bone; M, 1st tarsal bone; 2a, medial digital extensor tendon; 2b, common digital extensor tendon; 5a, LDE tendon ; 8, SDFT; 9, medial limb of LPL; 9a, lateral limb of LPL; 10, DDFT; 11, short digital extensor muscle; 13, long LCL; 15, long MCL; 24, inter-tarsal ligament; 31, plantar tarsal sheath; 32, short dorsal ligament; 33, medial plantar artery and vein

**Fig. 11:**
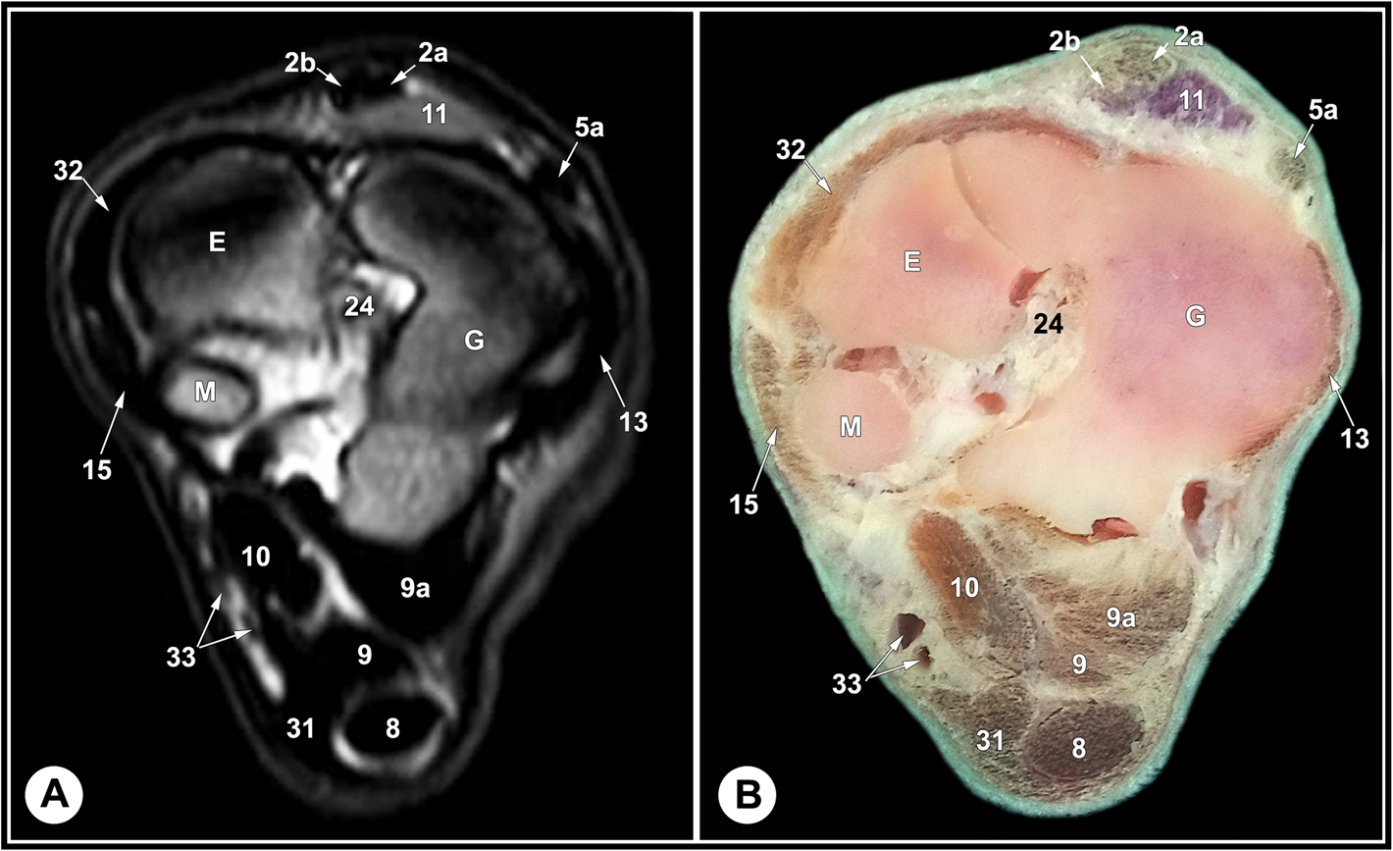
3D CT reconstructed dorsolateral view of the left dromedary camel tarsus showing the approximate levels of the selected MRI and gross sagittal (1–2), dorsal (3–4) and transverse (5–8) sections. A, distal tibia; B, calcaneus; B1, calcaneal tuber; C, talus; D, central tarsal bone; E, fused 2nd and 3rd tarsal bone; F, 3rd metatarsal bone; G, 4th tarsal bone; H, 4th metatarsal bone; J, malleolar bone; K, medial malleolus

None of the limbs showed tarsal abnormalities on the preliminary radiographic and ultrasonographic examinations. The osseous and soft tissue structures of the dromedary camel tarsus were illustrated (Figs. 1 and 2), and labeled in the MRI images and gross sections (Figs. 3, 4, 5, 6, 7, 8, 9 and 10). The level of each figure was indicated in Fig. 11. The use of high field MRI resulted into high quality images and various anatomical structures in the MRI images were identified and verified thanks to the gross anatomic sections. The overall time of the imaging protocol was around 40 minutes. T1 and PD-weighted images provided high anatomic details and allowed a thorough evaluation of articular cartilage and the peri-articular structures. T2-weighted and STIR sequences provided better visualization of synovial fluid.

The identified bony structures included: the tibial cochlea; calcaneus; talus; central, first, fused second and third, and the fourth tarsal bones; and the proximal metatarsal bone. Articular cartilage appeared as a layer of homogenous intermediate signal intensity on T1 images and had low signal intensity on T2 and PD images, with a smooth osteochondral junction. The cartilage surface was clearly defined in the tarsocrural joint and thin in the inter-tarsal and tarsometatarsal joints due to narrow joint spaces. The subchondral bone plate and cortical bone had homogeneous low signal intensity and cancellous bone expressed heterogeneous signal intensity with a well-defined trabecular pattern. The subchondral bone could be defined from articular cartilage and cancellous bone; however, it was not possible to define a boundary between the cortical bone and subchondral bone on the PD, T1, and T2 images, due to similar signal intensity (Figs. 3, 4 and 5, and 6).

The relevant soft tissue structures evaluated in the MRI images included the tendons of fibularis tertius, long digital extensor and cranial tibial muscles dorsally; the fibularis longus and lateral digital extensor muscles laterally; the caudal tibial and the lateral and medial digital flexor muscles medio-plantarly; and the long plantar ligament, the superficial (SDFT) and deep digital flexor tendons (DDFT) on the plantar aspect of the joint. Tendons were best evaluated in the transverse plane and had homogenous low signal intensity on all sequences (Figs. 7, 8 and 9, and 10). The SDFT, DDFT, and the medial and lateral limbs of the long plantar ligament were best visualized in the sagittal plane (Figs. 3 and 4). Synovial fluid had high signal intensity on STIR, PD, and T2 images and intermediate signal intensity on the T1 images (Figs. 3, 4 and 5, and 6).

The tarsal collateral ligaments consisted of short and long bundles on the medial and lateral aspects of the joint (Fig. 2). The long medial collateral ligament originated from the medial tibial malleolus, attached to the medial tarsal bones, and inserted into the medial proximal end of the metatarsus. The short medial collateral ligament consisted of three parts: a part connecting tibia and talus (tibiotalar branch); a part connecting tibia and calcaneus (tibiocalcaneal branch); and a wide part connecting talus and metatarsal bone. The long lateral collateral ligament originated from the malleolar bone, attached to the lateral tarsal bones, and inserted into the proximal metatarsus. The short lateral collateral ligament consisted of two parts: a part connecting the tibia and calcaneus (tibiocalcaneal branch) and a part between the base of calcaneus and metatarsal bone (calcaneometatarsal branch). The collateral ligaments were best evaluated in dorsal plane and had homogeneous low signal intensity in all sequences (Figs. 5, 6, 7, 8 and 9, and 10). The inter-tarsal ligaments connecting the talus and calcaneus and connecting tarsal bones had heterogeneous signal intensity (Figs. 5 and 6).

## Discussion

To the authors’ knowledge, this is the first published report providing description of the high field MRI appearance of the normal dromedary camel tarsus. The signal intensity of the clinically relevant osseous and soft tissue structures of the dromedary camel tarsus was described and the MRI images corresponded well with the gross anatomic sections. The obtained information in the current study appointed that MRI enabled assessment of structures inside the tarsus (soft and bony tissues) that otherwise cannot be imaged by other means and offers the opportunity to diagnose lesions within the tarsus that cannot be investigated through other imaging modalities [18]. MRI permitted viewing of the camel tarsus in three planes and obtaining information of cartilage, cortical bone, subchondral bone, trabecular bone, cancellous bone, ligaments, and tendons which is not all possible with other available diagnostic imaging tools. This enables the clinicians to interpret the tarsus in different angles and accurately detect the problem. The obtained results are in agreement with the conclusions reported earlier, that MRI offers the best evaluation technique of all anatomical structures, particularly soft tissues, of the tarsal joint in horse, cattle, dog and cat [3, 12–16].

The camel tarsus (hock) is a composite joint made up of four articulations: the tarsocrural, proximal intertarsal, distal inter-tarsal, and the tarsometatarsal joints. The tarsal joints in horses, cattle and camels share some similarities; however, numerous intra-articular and peri-articular anatomical variations exist among those species. The hock joint consists of six tarsal bones in camel and horse and five tarsal bones in cattle. However, the pattern of tarsal bone arrangement is variable: in camels, the second and third tarsal bones are fused; in horses, the first and second tarsal bones are fused; and in cattle, the central and fourth and the second and third tarsal bones are fused. Moreover, the distal part of fibula in camels and cattle persists as an isolated bone (malleolar bone), which articulates with the distal extremity of the tibia, while in horses, it is completely fused to the tibia and forms the lateral malleolus. Furthermore, the talus in camels and cattle: bears a trochlea at either end; the proximal trochlear ridges are directed sagittaly; and the distal trochlea is well defined and articulates with the fused central and fourth tarsal bones in cattle and with the central and fourth tarsal bones in camel. In horses, the talus bears one proximal trochlea and the trochlear ridges are orientated obliquely in a mediolateral direction. The calcaneus in camels and cattle articulates with the distal tibia, while in horses, it has no articular surface for the tibia and covers less of the lateral aspect of the talus than in cattle and camels. Regarding the periarticular soft tissues, the tarsus of cattle and camels have more tendons and ligaments than horses: the fibularis longus tendon; an additional bundle of the short medial collateral ligament connecting the talus and the medial metatarsal bone; and the split of the long digital extensor tendon to the common extensor of digits III and IV and the medial digital extensor. In camels also, the long plantar ligament consists of a medial and lateral limbs, unlike cattle and horses in which the ligament is undivided [1, 19–21]. The anatomical differences among camels, cattle and horses are likely to direct the attention towards demonstration of the magnetic resonance appearance of various intra-articular and peri-articular structures of the dromedary camel tarsus.

In this study, the spin-weighted sequences (T1, T2, PD and STIR) provided high anatomic definition and good tissue contrast in the dromedary camel tarsus. The T1 and PD images were appropriate for the detailed anatomic assessment of tarsal structures and the STIR and T2 sequences were valuable for investigation of the synovial fluid [5, 12, 19]. The protocol demonstrated in the present study was designed to optimize evaluation of various structures in the dromedary camel tarsus, although it was longer than that expected in clinical patients. This was to afford comprehensive reference images of the clinically normal camel tarsus to assist interpretation of MRI images in the clinical situations. The used sequences were extensive in every plane so that an optimal protocol could be determined. Under clinical circumstances, shorter protocols are used in order to shorten the acquisition time and subsequently the cost and duration of general anesthesia.

Magnetic resonance imaging is frequently used in horses owing to its ability to produce high-contrast and anatomically detailed tomographic images [9, 22]. The use of MRI in camels is still in its infancy and was limited to cadaveric studies. Cadaver limbs are commonly used for evaluation of MRI anatomy and the signal intensity recorded in cadavers was similar to that reported in live animals [12] and findings would be generalizable for live animals. The present study provided the first ever conducted detailed description of the normal anatomy of the dromedary camel tarsus on MRI images. By knowing the variation in MRI images in the normal camel, it would be possible to understand the importance of signal intensity changes in the clinical patients. Additionally, description of the high field MRI appearance of the tarsal joint structures can also be useful in the interpretation of radiographs, ultrasound, and computed tomography of the camel tarsus [22].

Currently, the use of MRI in camels is overburdened by cost, necessity for general anesthesia, and limited availability of equipment. High field MRI scanners are now available at a small number of equine clinics in the Middle East (where camels exist in high populations) and are increasingly available and developing fast for veterinary use. We do believe that the use of MRI in camels is still a new and developing field and would be a useful diagnostic imaging modality in the future for evaluation of various dromedary orthopedic problems, besides that it is currently a valuable imaging modality for investigational purposes. As clinical availability of this modality increases in the future, it is expected that new orthopedic disorders concerning dromedary camels will be identified that have not previously been reported with other imaging modalities. In the present study, the camel tarsus was a good candidate for the high field MRI. Its narrow linear profile and minimal soft tissue coverage allowed the use of the human extremity coil and enabled close apposition of the magnetic field to the tarsus resulting into good signal intensity as reported for the tarsus of horses and cattle [12, 14]. General anesthesia in camels obligates particular approach due to the different anatomical and physiological features of this species [23]. Tracheal intubation is necessary whenever general anesthesia is selected; however, endotracheal intubation in camels is a challenge [24], especially in males, due to the presence of a diverticulum of the ventral aspect of the soft palate [1]. Hence, a guiding tube and a long laryngoscope are needed to accomplish successful endotracheal intubation [25]. Accordingly, understanding of the anatomical and physiological dissimilarities of camels is indispensable to ensure a successful outcome of anesthesia and MRI examination in this species.

Knowledge of the normal anatomy and signal intensity of various tissues are crucial for correct interpretation of MRI scans obtained from lame patients. In the present study, synovial fluid had high signal intensity in STIR, PD and T2 images and intermediate signal intensity in the T1- weighted images and it was not possible to define a clear limit between the subchondral and cortical bones as both had low signal intensity. Similar findings were reported in the horse [3, 13]. The corticocancellous junction was regular and clearly defined, as mentioned for the bovine tarsus [14]. Cancellous bone had heterogeneous intermediate to high signal intensity where the bone of the trabeculae had low signal intensity and fat in the bone marrow filling the trabecular spaces had high signal intensity. This agreed with those reported in the horse [13]. Articular cartilage had homogenous intermediate signal intensity, on T1 images, adjacent to the low signal of subchondral bone at articular interfaces. Similar findings were reported in the horse [26]. The tarsocrural joint capsule, synovial tissue and synovial fluid had low to intermediate signal intensity similar to those reported in the bovine tarsus [14]. Tendons, collateral ligaments and the long plantar ligament of the dromedary camel had low signal intensity and the inter-tarsal ligaments had heterogeneous signal intensity. Similar findings were reported in horse [10, 27].

Diagnostic imaging continues to play an important role in the assessment of joint injuries and assists investigators to understand the risk factors associated with the onset and progression of the disease condition. Conventional radiography is the simplest, least expensive and most commonly deployed imaging modality over the last decades because of its reproducibility and feasibility to detect structural damage; however, radiography can provide only indirect information on soft tissues and insensitive to early inflammatory bone involvement and bone damage. [8]. Ultrasonography enables real-time imaging of synovial pathology, articular cartilage and cortical erosive changes at relatively low cost but it is an operator-dependent and the physical properties of sound limit its ability to assess deeper structures [28]. Diagnostic arthroscopy is a crucial skill for diagnosing intra-articular disorders as it enables a direct magnified view of the cartilage surface. However, it is an invasive method, diagnosis is based only on subjective visual evaluation and manual mechanical palpation and deeper joint structure are inaccessible due to anatomic limitations [29]. MRI has become a key imaging tool for evaluation of joint pathology thanks to its ability to assess pathologic and biochemical changes within the joint before morphologic changes become evident with conventional imaging tools. In addition, the multi-plane and multi-slice capability of MRI enables visualization of the area of interest in three orthogonal planes. Therefore, MRI has the advantage of providing details concerning bone, articular cartilage and peri-articular structures, which is not shared by any other imaging modalities [4].

High field MRI provided comprehensive assessment of the dromedary camel tarsus. Results of the present study indicated that signal intensity varied according to the used sequence and investigated structure in the normal dromedary camel tarsus. Interpretation of MRI images is a challenge and obliges a good knowledge of anatomic details and familiarization to the normal MRI appearance of the region of interest in order to diagnose the problem with confidence. Further studies are warranted to determine the clinical application of high field MRI in the dromedary camel tarsus.

## Conclusions

This study demonstrates and supports the use of high field MRI to evaluate various components of the dromedary camel tarsus and have the potential to become a useful reference for interpretation in clinical situations.

## Methods

### Animals

Six adult dromedary camels (four males and two females) were referred to the Veterinary Teaching Hospital, College of Veterinary Medicine, King Faisal University. The age of animals ranged from 6 to 14 years (mean ± SD, 8 ± 2.5) and weight from 480 to 700 kg (mean ± SD, 594 ± 93). Camels were euthanized for reasons unrelated to the study or orthopedic problems. Immediately after euthanasia, the right and left hind limbs of each camel were disarticulated at the stifle joint, to maintain normal soft tissue tension, and examined within two hours. Prior to MRI, Tarsi were examined radiographically in the four standard planes followed by a thorough ultrasonographic examination in both horizontal and longitudinal planes, with the purpose of screening for potential abnormalities.

The study was a prospective cadaveric study. A sample size of 12 was determined based on this being a novel study concept, as this value represents the first significant increase in power in a sample size for a study where no previous datasets were available as a reference on which to base calculations [22].

### MRI protocol

Immediately after the preliminary imaging investigations, limbs were positioned with the lateral aspect contacting the MRI table and foot entered the magnet first to replicate clinical positioning. MRI images were acquired using a human extremity coil and a high field 1.5 Tesla magnetic resonance scanner (Philips Ingenia 1.5T MRI; Philips GmbH, Hamburg, Germany).

Three-plane scout images (transverse, sagittal and dorsal planes) were obtained to ensure correct positioning of the specimen and proper orientation of the following sequences. The transverse plane was oriented perpendicular to the long axis of the calcaneus. The sagittal plane was defined parallel to the sagittal plane of the calcaneus. The dorsal plane was perpendicular to the sagittal and transverse planes, aligned with the plane of the metatarsal bone. Tarsi were scanned via Turbo Spin-echo (TSE) sequences in T1- weighted (T1), T2-weighted (T2), proton density-weighted (PD), and short tau inversion recovery (STIR) sequences in the transverse, sagittal and dorsal planes. The acquisition settings used for the MRI scanning of the camel tarsus are displayed in Table 1.

**Table 1:** Scanning parameters of the magnetic resonance imaging sequences used in the study

	T1W TSE	T2W TSE	PD	STIR
**Slice thickness (mm)**	4	4	4	4
**Gap width (mm)**	1	1	1	1
**Matrix**	256 × 256	256 × 256	256 × 256	256 × 256
**TR (repetition time; milliseconds)**	655	3100	3650	4800
**TE(echo time; milliseconds)**	7	60	30	25
**Flip angle**	90	90	90	90
**Field of view (cm)**	24	24	24	24

### Subjective evaluation of the MRI and gross images

After MRI, limbs were frozen at -18˚ C for one week. Each 4 tarsi were randomly selected, sectioned (thickness, 1 cm) and photographed. The slice number per tarsus was 35, 18 and 14 slices in the transverse, dorsal and sagittal planes, respectively. A total number of 140 (transverse), 72 (dorsal) and 56 (sagittal) slices were enrolled in the study. MRI images were assessed to establish and record the normal high field MRI appearance and signal intensity for each structure. MRI images were then compared to their corresponding cryosections. The most representative MRI images at various levels that were best correlated with the macroscopic slices were selected.

## Data Availability

The datasets used and/or analysed during the current study are available from the corresponding author on reasonable request.

## Abbreviations

MRI: Magnetic resonance imaging
TSE: Turbo Spin-echo
T1: T1-weighted
T2: T2-weighted
PD: Proton density
STIR: Short tau inversion recovery
SDFT: Superficial digital flexor tendon
DDFT: Deep digital flexor tendon

## **Authors’ information**

Dr. Zakriya Ali Almohamad and Dr. Usama Hagag are joint first authors.
